# Supplementary figures and images for: Mapping the degradation pathway of a disease-linked aspartoacylase variant
Source: PLoS Genet. 2021 Apr 29;17(4):e1009539. doi: 10.1371/journal.pgen.1009539 (PMC8084241; doi:10.1371/journal.pgen.1009539)

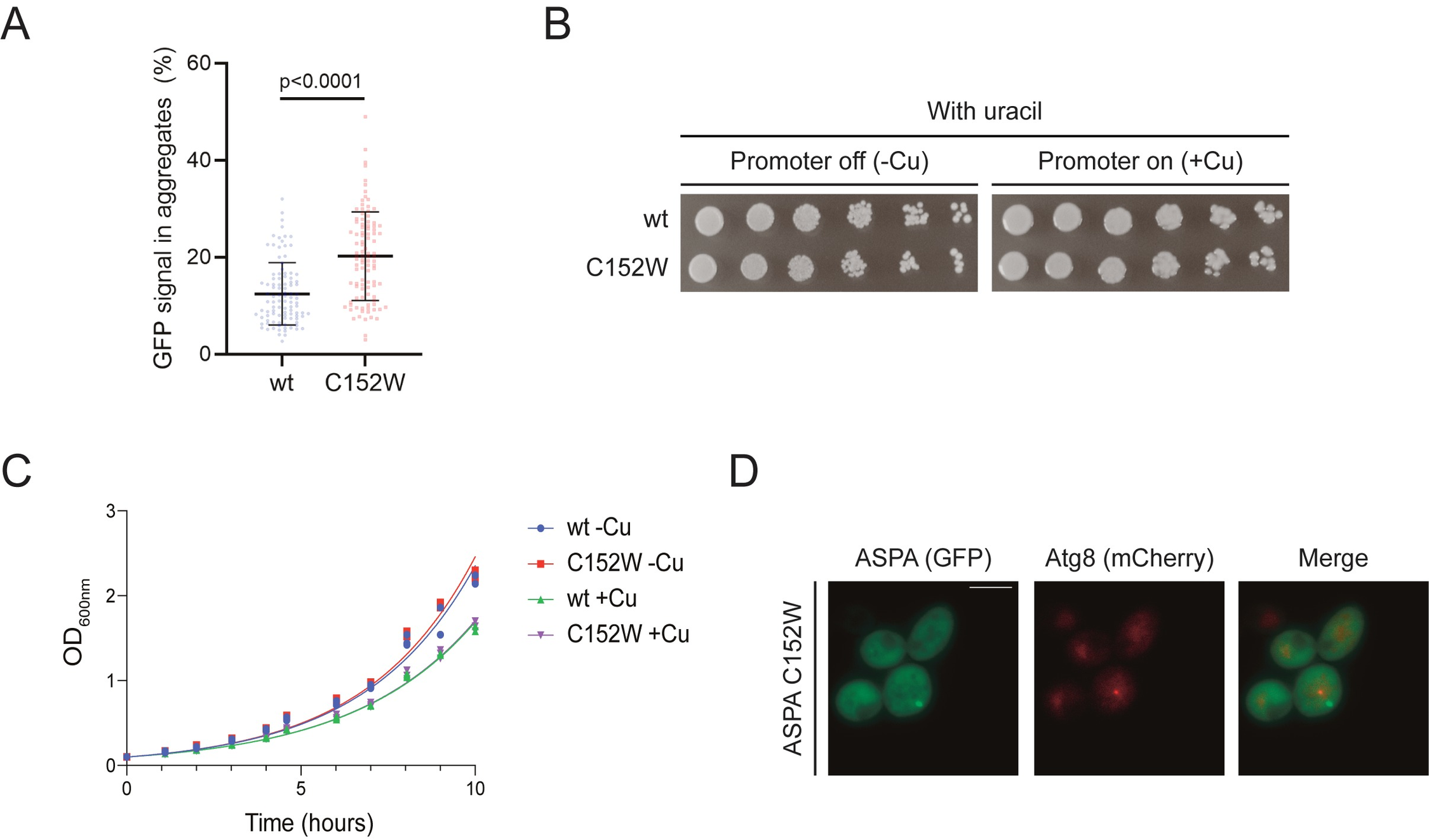

Supplement: S1 Fig — (A) The percentage of total GFP signal found in aggregates in individual cells expressing either wild-type ASPA or C152W was quantified in 100 cells. Each data point represents the signal distribution in a single cell. The GFP signal found in aggregates was compared between wild-type ASPA and C152W using Welch’s t test. Means and standard deviations are shown. (B) Wild-type yeast cells expressing either wild-type ASPA or the C152W variant grown on solid medium containing uracil and without or with copper as indicated. (C) Wild-type yeast expressing the indicated constructs were grown in liquid medium containing uracil without or with copper as indicated, and the OD600nm was measured at the indicated time points. Growth was compared using Tukey’s multiple comparisons test on three independent cultures for each condition. There was no significant difference between the doubling time of wild-type ASPA or the C152W variant in absence nor in presence of copper. The doubling times in absence and presence of copper were significantly different (adjusted p-value < 0.0001, n = 3) possibly caused by copper itself or the high expression level of an exogenous protein. (D) Representative fluorescence microscopy images of wild-type yeast expressing GFP-ASPA C152W and mCherry-Atg8. Only rarely did we observe co-localization between ASPA aggregates and Atg8, indicating that aggregated ASPA C152W does not accumulate in the IPOD [49]. Scale bar is 4 μm. (TIF) [file pgen.1009539.s001.tif]

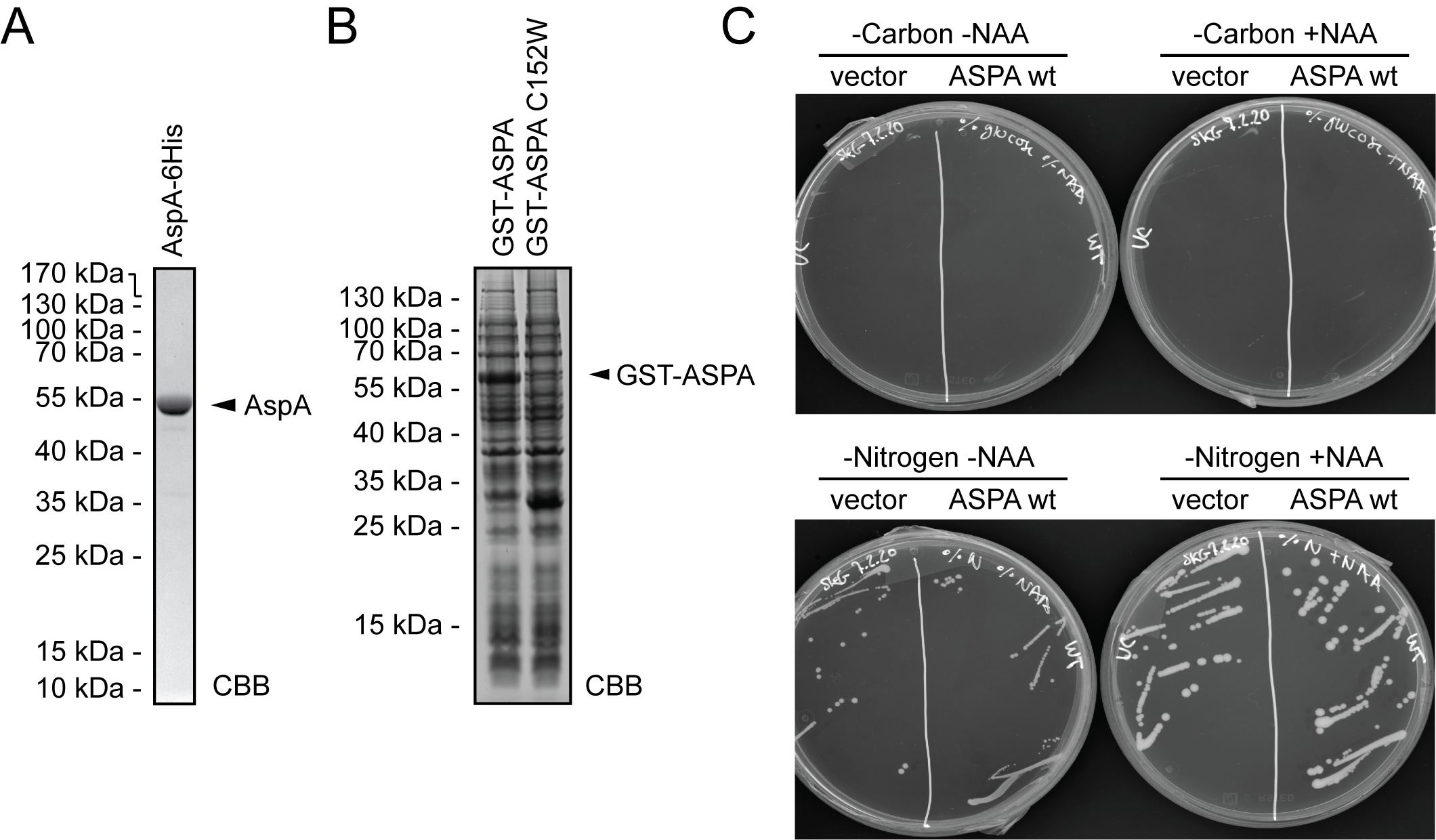

Supplement: S2 Fig — (A) As a helper enzyme to convert produced aspartate into fumerate for an ASPA enzyme assay [7], 6His-tagged E. coli AspA was produced in E. coli and purified. A sample of the purified material was resolved by SDS-PAGE and the gel was stained with Coomassie Brilliant Blue (CBB). (B) ASPA and ASPA C152W was produced in E. coli fused N-terminally to GST. The material was resolved by SDS-PAGE and the gel was stained with Coomassie (CBB). (C) Wild type yeast cells transformed with a control plasmid (vector) or an expression plasmid for wild-type ASPA were streaked as indicated on minimal media plates where either the carbon source (upper panel) or the nitrogen source (lower panel) was exchanged with N-acetylaspartate (NAA). (TIF) [file pgen.1009539.s002.tif]

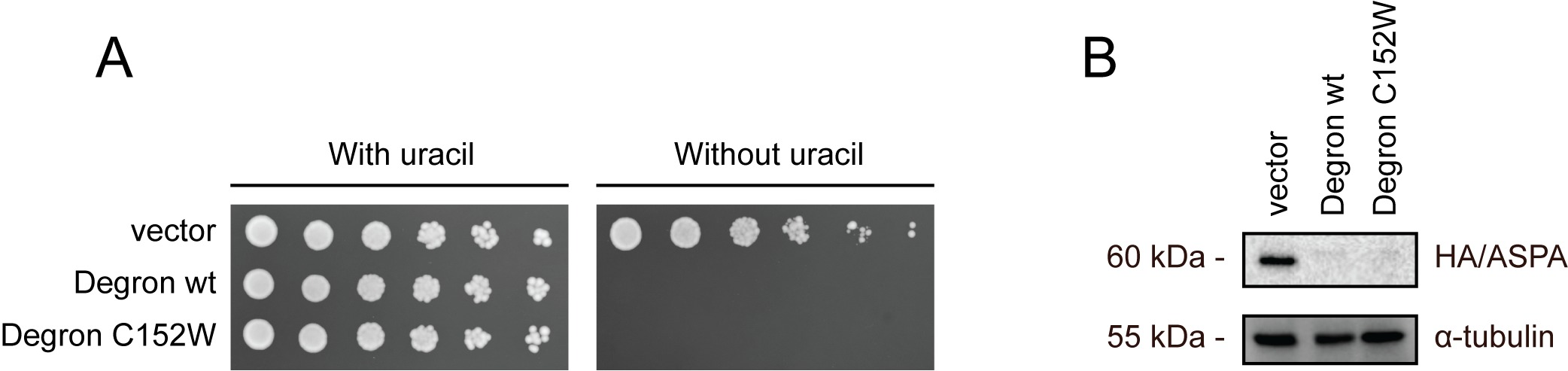

Supplement: S3 Fig — (A) Growth of wild-type yeast cells expressing an empty vector control or potential degrons consisting of 28 amino acids surrounding the C152 residue in the ASPA sequence without (Degron wt) or with (Degron C152W) the C152W substitution. (B) Western blot showing the protein levels of the degron constructs. Tubulin served as a loading control. (TIF) [file pgen.1009539.s003.tif]

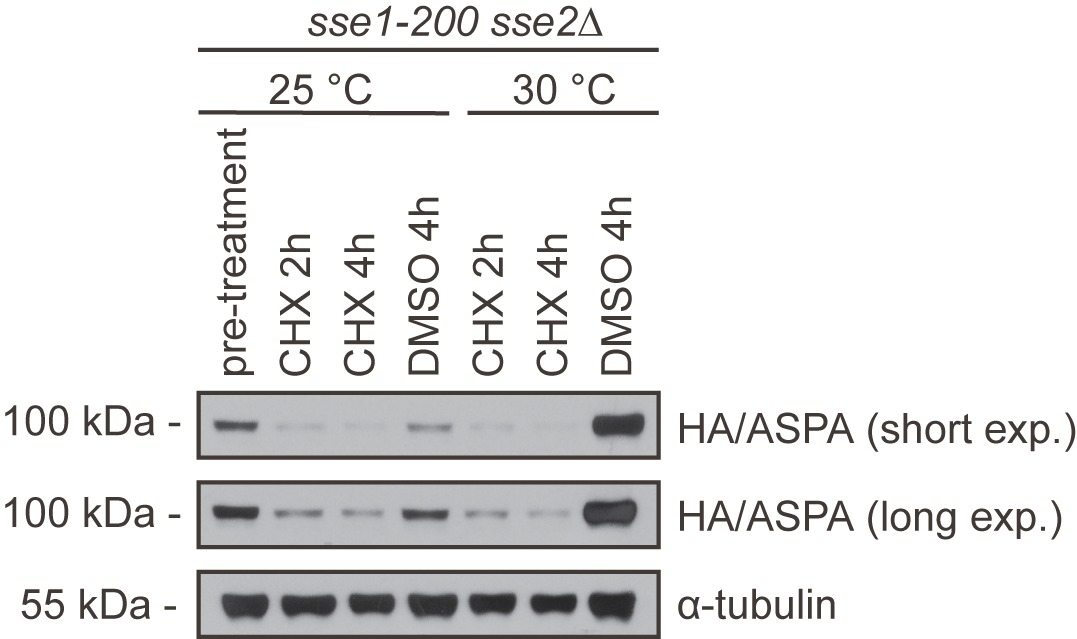

Supplement: S4 Fig — The sse1-200 sse2Δ strain expressing ASPA C152W was incubated at 25°C until exponential phase. The culture was then split and cells were incubated at the permissive temperature (25°C) or restrictive temperature (30°C) with the translation inhibitor cycloheximide (CHX) or as a control the solvent DMSO. Cells were harvested at the indicated time points, followed by protein extraction and Western blotting to examine the steady state of ASPA C152W. Tubulin served as a loading control. (TIF) [file pgen.1009539.s004.tif]

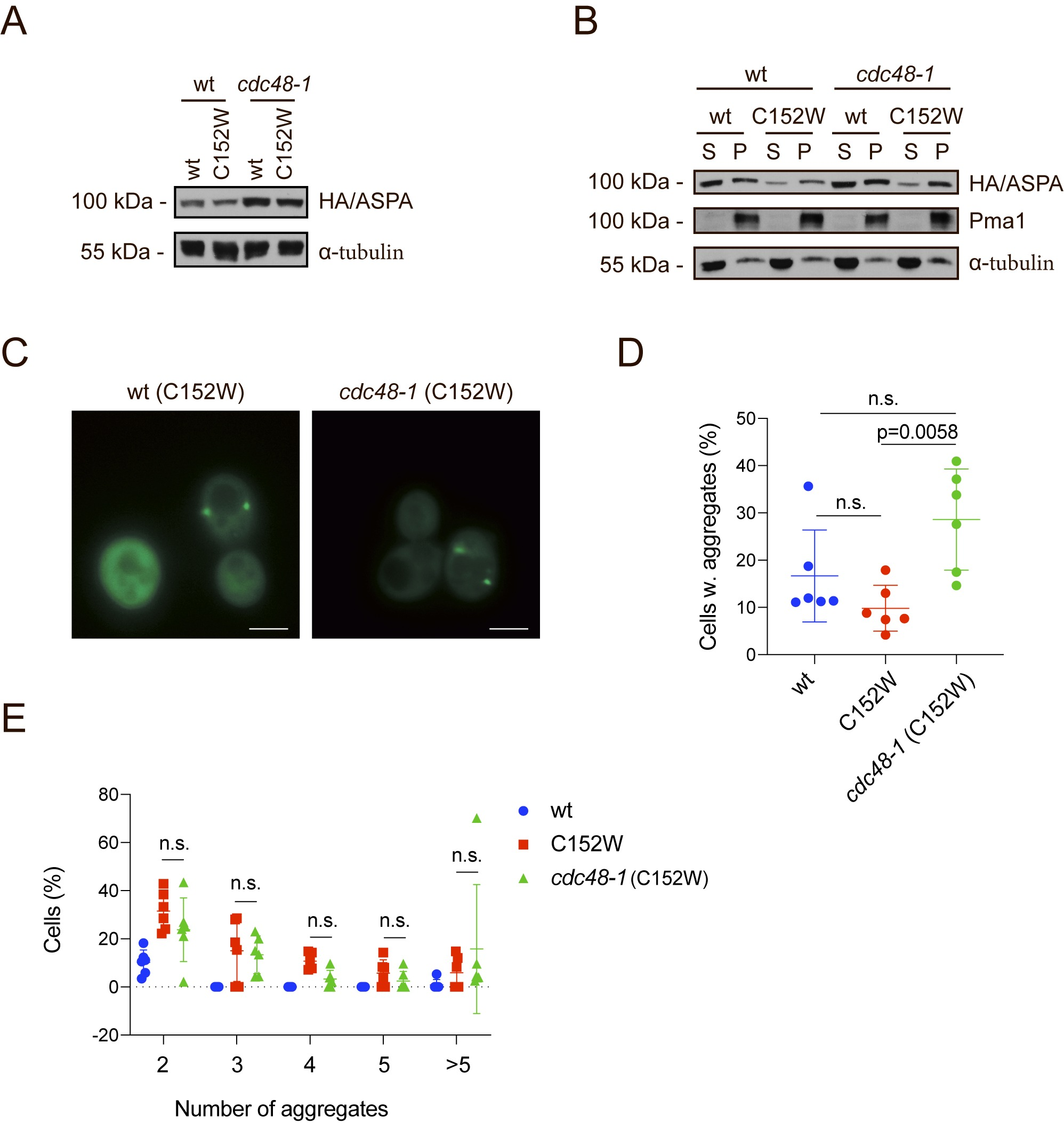

Supplement: S5 Fig — (A) Western blot of ASPA protein levels in wild-type and cdc48-1 yeast strains expressing the indicated constructs cultured with 0.1 mM CuSO4. Tubulin served as a loading control. (B) Western blot showing the levels of soluble (S) and insoluble (P) ASPA in wild-type and cdc48-1 yeast strains expressing the indicated constructs in presence of 0.1 mM CuSO4. Tubulin and Pma1 served as controls for the soluble and insoluble fractions, respectively. (C) Representative fluorescence microscopy images of GFP-ASPA C152W in a wild-type or cdc48-1 strain. Scale bars are 4 μm. (D) Cells expressing GFP and containing aggregates were counted using fluorescence microscopy of wild-type (wt, C152W) and cdc48-1 yeast strains expressing the indicated constructs. Data points indicate frequencies from independent experiments (minimum of 127 GFP-expressing cells, n = 6). The frequencies of cells containing aggregates were compared using a one-way ANOVA and Tukey’s multiple comparisons test in GraphPad Prism. Means and standard deviations are shown. (E) Cells containing aggregates and the number of aggregates were counted by fluorescence microscopy of wild-type (wt, C152W) and cdc48-1 yeast cells expressing the shown constructs. The frequencies of each observation in separate experiments are shown as data points (minimum of 127 GFP-expressing cells, n = 6). The aggregate patterns were compared using a two-way ANOVA and Tukey’s multiple comparisons test. Means and standard deviations are shown. (TIF) [file pgen.1009539.s005.tif]

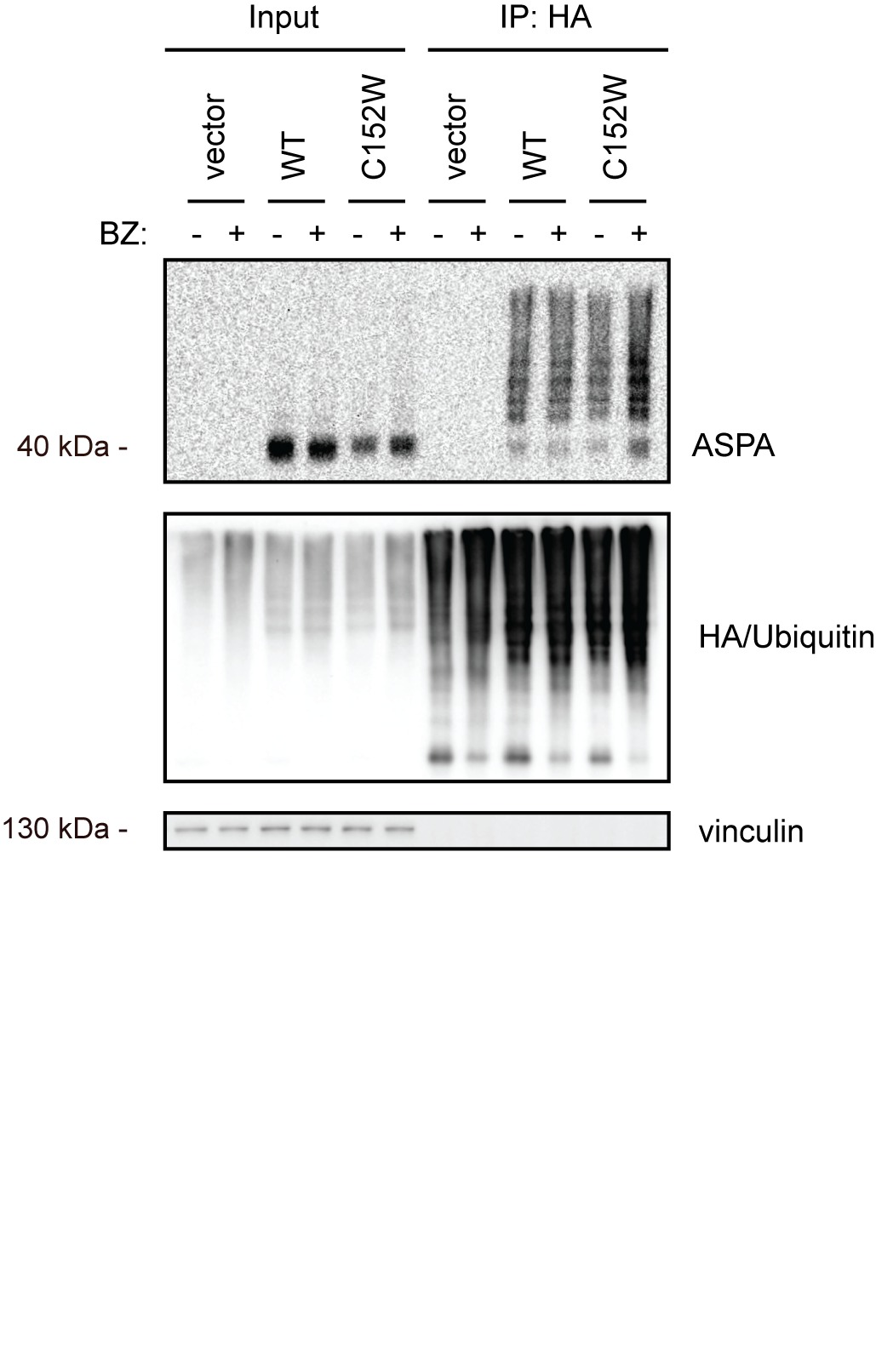

Supplement: S6 Fig — U2OS cells transiently transfected to express HA-ubiquitin and a vector control, wild-type ASPA or C152W were treated with bortezomib (BZ, +) or as a control DMSO (-) followed by denaturing HA immunoprecipitation. Vinculin serves as a loading control. Note that the level of ubiquitinated ASPA C152W increases following treatment with bortezomib. (TIF) [file pgen.1009539.s006.tif]

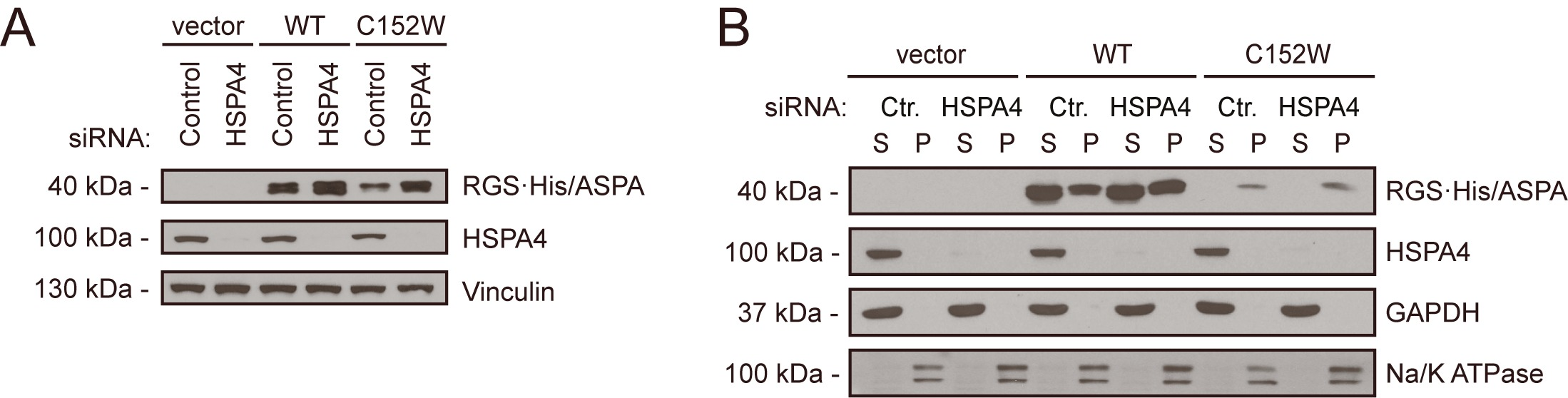

Supplement: S7 Fig — (A) Knock-down of HSPA4 in U2OS cells transiently transfected with the indicated constructs results in stabilization of ASPA. Successful knock-down was confirmed by blotting for HSPA4. Vinculin served as a loading control. (B) Cells were treated as in A, followed by differential centrifugation to separate soluble and insoluble protein into supernatant (S) and pellet (P) fractions. Successful knock-down was confirmed by blotting for HSPA4. GAPDH and the Na/K ATPase served as controls for soluble and insoluble proteins, respectively. Note that ASPA C152W is exclusively found in the insoluble fraction. (TIF) [file pgen.1009539.s007.tif]

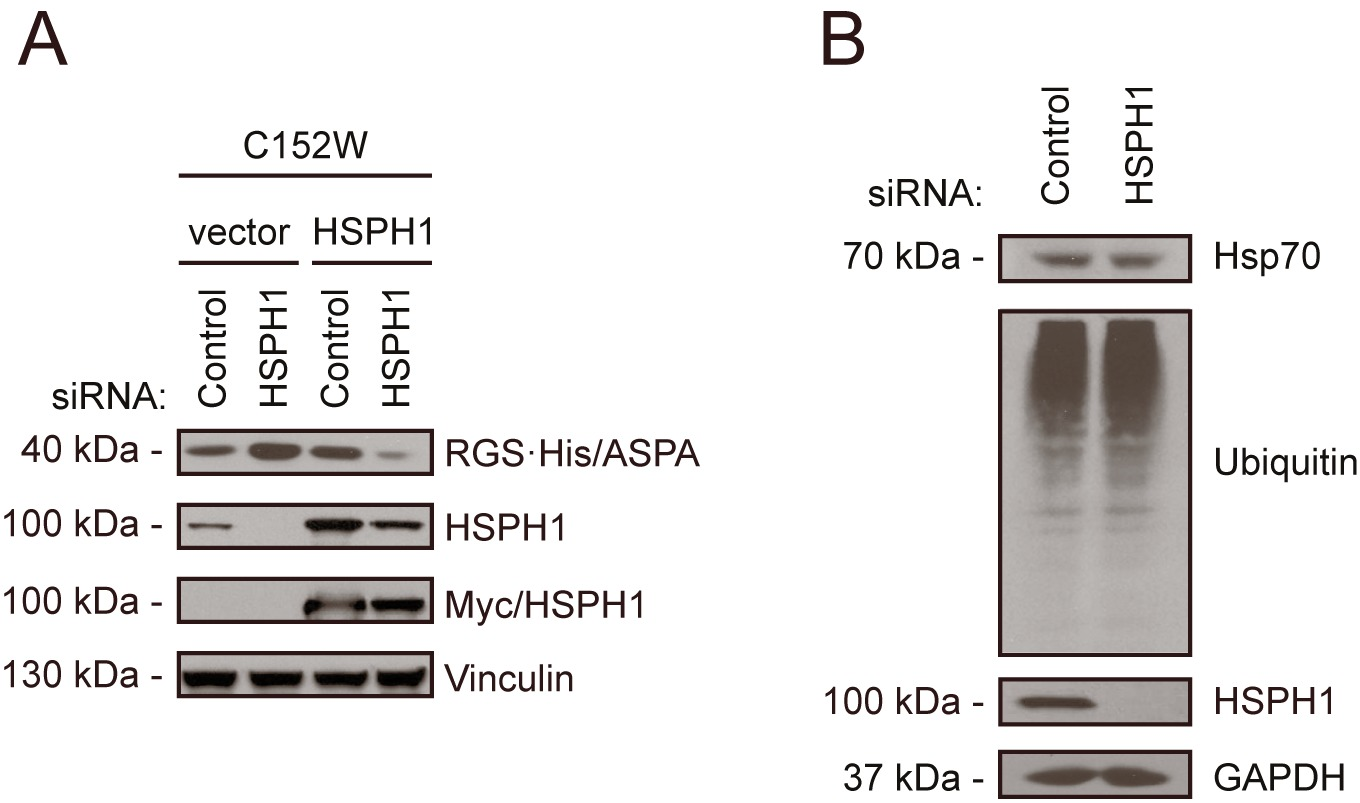

Supplement: S8 Fig — (A) U2OS cells transiently transfected to express ASPA C152W and HSPH1 or a vector control with and without HSPH1 knock-down. Note that expression of episomal siRNA-resistant myc-tagged HSPH1 restores degradation of ASPA C152W upon knock-down of endogenous HSPH1. Vinculin served as a loading control. (B) Knock-down of HSPH1 expression does not lead to detectable changes in the level of Hsp70 or ubiquitin-protein conjugates. GAPDH served as a loading control. (TIF) [file pgen.1009539.s008.tif]
