# Supplementary material for: Mapping the degradation pathway of a disease-linked aspartoacylase variant
Source: PLoS Genet. 2021 Apr 29;17(4):e1009539. doi: 10.1371/journal.pgen.1009539 (PMC8084241; doi:10.1371/journal.pgen.1009539)
Supplement: Attachment — Submitted filename: Response.docx [file pgen.1009539.s010.docx]

## Response to reviewers

Dear Dr. Tang and Dr. Williams

We thank you for your kind e-mail and excellent handling of our manuscript (PGENETICS-D-20-01411) submitted to PLOS Genetics. We were pleased to note that both reviewers found that our manuscript provides important new insights in protein quality control mechanisms and their relevance for a genetic disease. We have carefully read the reviewer comments and provide here an extensively revised version of our manuscript, including new data with protein aggregate quantifications presented in Fig. S1A. As described below and in the revised manuscript (e.g. new data in Fig. S2) we attempted to purify the C152W variant for enzymatic characterization, but unfortunately, this was not possible due to low expression level and poor solubility. We have therefore, as suggested by the reviewers, toned down our discussion of potential therapies targeting the PQC system, while maintaining a strong focus on our central observations on the protein quality control (PQC) regulation of the C152W variant.

Please find below our point-by-point response to the issues raised by the reviewers. In our opinion, the revision has substantially strengthened the manuscript, and we hope you will find it acceptable for publication in PLOS Genetics.

Finally, please note that we due to the additional experiments performed for the revision have added one author to the manuscript. All authors have approved the uploaded revised manuscript.

Yours sincerely

Rasmus Hartmann-Petersen

## Our point-by-point response to the raised issues

Major Revisions:
1. An essential experiment to substantiate the claims of this paper is an enzyme activity assay demonstrating that the rate of catalysis in the NAA to acetate + aspartate is not significantly perturbed due to the C152W mutation. Although the references cited show from crystal structures that the C152W mutation is outside the active site, in order to support the claim and investigation that C152W pathogenic mechanism is exclusively due to increased degradation, evidence of equivalent enzymatic activity is needed.

*Our response: Although the primary focus of our manuscript is on the PQC regulation of the C152W variant, we agree that this would be a nice addition to the paper. Unfortunately, however, it has proven to be complicated by the C152W variant being expressed at a low level and the protein being highly insoluble. In addition, we note that the published kcat for wildtype purified ASPA is 0.08 s^-1^ (PMID:* ***12706335****), hence even under optimal conditions, ASPA is not a very active enzyme, thus demanding highly concentrated preparations for enzymatic studies.*

*During the revision we have attempted to approach this in four independent ways: 1) By assaying yeast cell lysates we found that there was not enough wildtype ASPA activity to obtain a reliable activity estimate. 2) We found that it was not possible to purify C152W from cultured mammalian cells, since the protein is largely insoluble (see manuscript Fig 6F). 3) After codon optimization for production in E. coli we managed to produce some wildtype ASPA, but not significant amounts of C152W, even when fused to GST (to help folding and increase solubility) (Fig S2B). 4) Finally, we tested if we could generate an in vivo ASPA assay in yeast cells by substituting the carbon or nitrogen source with NAA. However, we found no ASPA-dependent difference in growth on media prepared with NAA as the only C-source or N-source (Fig S2C). Hence, in conclusion, it is possible that the C152W variant retains some enzyme activity, but we were unfortunately not able to test this directly and have included these results and conclusions in the manuscript (p. 9-10, l. 180-190, Fig S2). Accordingly, since we were unable to prove that the C152W mutation entirely causes disease due to PQC-linked degradation, we have changed the manuscript to reflect this point (p. 7 l. 134-137, p. 19 l. 388-391, p. 19-20 l. 410-412). As also suggested, we focus on the PQC aspects of our data using C152W as a model protein, and as pointed out by the reviewers below (point 4), we have throughout (p. 2 l. 40-42 and p. 7 l. 132-137, p. 19 l. 388-391) toned down the possibility of potential therapies targeting the PQC.*

*Finally, we note that while we were unsuccessful in obtaining sufficient amounts of C152W, others have previously found that this variant does not display significant activity (PMID: 17391648, 12638939, 22850825) when produced in Pichia or COS-7 cells. We now mention this in the manuscript (p. 19 l. 388-391).*

*Please note the all page & line numbers refer to the unmarked version of the manuscript.*

2. The aggregation experiments in figure 2 are not entirely convincing though providing additional data analysis may be sufficient and not necessarily a repeat of the experiment. In addition to the quantity of aggregates, the distribution, area, and intensity of the foci and diffuse protein should also be measured. From the representative IHC, it appears that WT is more diffuse throughout the cells than appearing in aggregates. This feature can be quantified and calculated as a ratio of diffuse:aggregate signal, and this should demonstrate that the majority of ASPA C152W appears in foci in contrast to diffuse through the cytoplasm.

*Our response: This is an important issue, and we thank the reviewers for pointing it out. We have now included a quantification of the percentage of GFP signal found in aggregates compared to the total GFP signal in each cell (p. 9 l. 170-171, Fig S1A). A higher percentage of GFP signal is found in aggregates for cells expressing ASPA C152W compared to wild-type ASPA, consistent with the fluorescence images and aggregate quantifications in figure 2, although the majority of ASPA C152W GFP signal is not found in foci.*

a. In addition, panel 2F and 2G can be merged by including 0 aggregates and 1 aggregate as data points.

*Our response: Since we use similar aggregate quantifications for wild-type, hsp104Δ and cdc48-1 cells, we prefer to keep panel 2F and 2G apart to make all the quantifications in the same format. For the cdc48 quantification, the similar aggregation pattern in cdc48-1 and wild-type cells would not be apparent if the quantifications were merged, as there are more cells with aggregates in cdc48-1 cells. However, we have made the two panels smaller to accommodate the previous panel S1C (see point 2b).*

b. Adding panel S1C to figure panel 2 would be desirable.

*Our response: This is a good point, we have now added former panel S1C to figure 2F.*

3. The degron experiment in Figure 3 C and D isn’t that helpful in supporting the claim that C152W exposes the degron during de novo folding. Since WT and C152W show equivalent results, it simply supports the claim that the sequence in and of itself contains a degron. But it does not show that C152W increases the accessibility/exposure of the degron; there needs to be a contrast. It might be useful to attempt an experiment eliminating the degron from ASPA or making sufficient amino acid substitutions to show that removing the sequence as a whole has a different effect than the C152W substitution.
a. Otherwise, consider relocating panel S2 to figure panel 3 and placing 3D in the supplement.

*Our response: We agree that experimental evidence supporting that C152W increases the accessibility/exposure of the degron is lacking. However, eliminating the degron would require substitution or deletion of multiple amino acid residues, making it difficult to interpret the results of such an experiment because substitutions would likely also change the overall stability and folding of the protein. As proposed, we have instead relocated former figure S2 to figure 3 and have placed former figure 3C and D in the new figure S3.*

4. The claim that HSP’s and PQC machinery be targeted for therapeutic treatment of the disease is rather bold. Although the authors concede that these protein networks have broad involvement among the degradation dynamics of many proteins, evidence that targeting Hsp110 and Hsp70 does not substantially affect other targets is necessary to put forth such a claim. The authors can instead rephrase these statements as a call to further investigation into the possibility of therapeutically targeting these proteins to treat disease.

*Our response: We agree and have rephrased the statements as suggested (p. 2 l. 40-42 and p. 7 l. 132-137).*

a. In relation to this point, some description of gene replacement and enzyme replacement therapies for Canavan Disease should be provided in greater detail. The authors could explain that this research focuses on to addressing misregulated degradation dynamics. (i.e. explain that this research relates to addressing protein misfolding diseases as a whole and Canavan Disease simply provides a useful context for this research). It would be helpful to mention other protein misfolding diseases that this research can be applied to. However, there is no definitive evidence among this research that Hsp70 and Hsp110 are indisputable therapeutic targets and therefore such a claim needs to be minimized.

*Our response: We agree with the suggestions and have now included a description of the currently investigated therapies for Canavan disease (p. 4 l. 77-81). In addition, we have emphasized our focus on altered degradation dynamics (p. 5 l. 90-92) and have included examples of other protein misfolding diseases for which this research might be relevant (p. 20 l. 415-416). The claim of Hsp70 and Hsp110 as therapeutic targets has been minimized (see point 4).*

b. The most useful conclusions from this research are that subtle rate changes in de novo folding can expose degrons during an intermediate state which subsequently accelerates degradation depleting essential metabolic enzymes. This in and of itself can result in a disease phenotype. Therefore, the focus of the manuscript ought to underscore the importance of investigating protein folding, degradation dynamics, and deconvolution of the pathway to degradation (Not the disease).

*Our response: We agree that the most useful findings in our study are related to protein degradation dynamics and pathways, and have included sentences to underscore the use of ASPA C152W as a model of a disease-causing protein variant with an increased cellular turnover (p. 2 l. 28-29, p. 5 l. 90-92).*

Minor Revisions:
1. Recommendation to modify title to reflect a conclusion of the research as opposed to a general topic. (e.g. Increased proteasomal degradation of disease-linked aspartoacylase variant is a major pathogenic mechanism of disease progression)

*Our response: We have modified the title to reflect better the focus of our research, which is the deconvolution of the pathway to degradation and not the disease in accordance with point 4b:*

*“Mapping the degradation pathway of a disease-linked aspartoacylase variant”.*

2. Recommendation to better clarify the difference between ASPA’s native folding and structure vs. the process of de novo folding. For example, in the abstract, “…indicating that the native state is structurally preserved” and “…prevents ASPA from reaching its stable native conformation” seem conflicting upon first read. Perhaps clarify the latter sentence to: “C152W substitution interferes with the de novo folding process resulting in increased proteasomal degradation before reaching its stable conformation”.

*Our response: We agree and have made the distinction between structure and de novo folding clearer (p. 2 l. 32-34, p. 6 l. 127-128).*

3. The sentence starting on line 155 can be rewritten as a comparison between C152W and WT. (e.g. “While WT ASPA was mainly soluble, the C152W variant, in contrast, demonstrated increased insolubility”)

*Our response: We have adapted the text as suggested (p. 9 l. 165-166).*

4. The figure legend of 3A and related text should include the software used to produce the figures despite describing in the methods section.

*Our response: We thank Reviewer 2 for pointing this out. We have now included the software in the figure legend (p. 40 l. 959-960) and related text (p. 10 l. 197-198).*

5. I didn’t quite follow the logic in jumping immediately into studying HSP70. Perhaps substantiating the particular decision to investigate Hsp70 would be helpful to the text narrative. What other proteins were considered for analysis and why was Hsp70 chosen for careful investigation?

*Our response: This is a great suggestion and accordingly we have clarified the central role of Hsp70 in mediating proteasomal degradation of destabilized and misfolded proteins (p. 11 l. 226-228).*

6. The illustration in figure 4A could be more detailed to help with a more general readership, e.g. labeling the proteasome.

*Our response: We have labeled the proteasome in figure 4A.*

7. The text regarding Cdc48 is a bit unclear. Perhaps including Cdc48’s mechanism in the illustration of panel 5A can better describe the involvement in the overall degradation pathway. It should also be clarified as to how Cdc48 relates to Hsp104.

*Our response: This is a great suggestion, and we have included Cdc48 in figure 5A, and clarified how Cdc48 relates to Hsp104 (p. 13 lines 268-270).*

8. At times it seems the word “stability” is conflated with “reduced aggregation/degradation propensity”. Please clarify the difference between protein folding (stability) and degradation dynamics (rate).

*Our response: We agree and have altered the text to make this distinction more clear (p. 3 l. 49-50, p. 6 l. 125, p. 12 l. 248-249, p. 14 l. 292, 294, 297-298).*
